# Diet-induced obesity impairs muscle satellite cell activation and muscle repair through alterations in hepatocyte growth factor signaling

**DOI:** 10.14814/phy2.12506

**Published:** 2015-08-21

**Authors:** Donna M D’Souza, Karin E Trajcevski, Dhuha Al-Sajee, David C Wang, Melissa Thomas, Judy E Anderson, Thomas J Hawke

**Affiliations:** 1 Departments of Pathology & Molecular Medicine, McMaster UniversityHamilton, Ontario, Canada; 2 Department of Biological Sciences, University of ManitobaWinnipeg, Manitoba, Canada

**Keywords:** Diet-induced obesity, growth factors, muscle repair, satellite cell

## Abstract

A healthy skeletal muscle mass is essential in attenuating the complications of obesity. Importantly, healthy muscle function is maintained through adequate repair following overuse and injury. The purpose of this study was to investigate the impact of diet-induced obesity (DIO) on skeletal muscle repair and the functionality of the muscle satellite cell (SC) population. Male C57BL/6J mice were fed a standard chow or high-fat diet (60% kcal fat; DIO) for 8 weeks. Muscles from DIO mice subjected to cardiotoxin injury displayed attenuated muscle regeneration, as indicated by prolonged necrosis, delayed expression of MyoD and Myogenin, elevated collagen content, and persistent embryonic myosin heavy chain expression. While no significant differences in SC content were observed, SCs from DIO muscles did not activate normally nor did they respond to exogenous hepatocyte growth factor (HGF) despite similar receptor (cMet) density. Furthermore, HGF release from crushed muscle was significantly less than that from muscles of chow fed mice. This study demonstrates that deficits in muscle repair are present in DIO, and the impairments in the functionality of the muscle SC population as a result of altered HGF/c-met signaling are contributors to the delayed regeneration.

## Introduction

In addition to our physical capacities, skeletal muscle also provides a major contribution to our whole-body metabolic control by regulating blood glucose and fatty acid (FA) levels through uptake, followed by utilization and/or storage. In fact, skeletal muscle comprises ∼40% of our body weight (in non-obese subjects), accounts for approximately 1/3 of our resting oxygen uptake, and is the site for up to 90% of our exercising oxygen uptake (Zurlo et al. 1990). Given the extensive contribution of skeletal muscle to our whole-body metabolic capacity, one can appreciate that in individuals who have a reduced relative muscle mass, lead a sedentary lifestyle, and/or suffer from a chronic illness, there would be a negative impact on their basal metabolic rate and their capacity to adequately manage circulating lipids and glucose (Wolfe 2006).

Our laboratory has previously demonstrated initial positive adaptations in skeletal muscle with high-fat feeding, followed by a decline in muscle health and the development of muscle insulin resistance (Shortreed et al. 2009; Trajcevski et al. 2013; Thomas et al. 2014). Additionally, previous literature using various models of obesity has already established that a number of unfavorable changes occur within skeletal muscle of obese rodents (for review, see Akhmedov and Berdeaux 2013). Elucidating the response of skeletal muscle to stimuli that promote damage and/or injury is relevant in the study of skeletal muscle health in obesity. Skeletal muscles from obese samples demonstrate an increased susceptibility to damage, as identified using both human (Salvadori et al. 1992) and animal (Knoblauch et al. 2013) models of obesity. As a result of this, diet-induced obesity is often associated with an atrophic rather than hypertrophic response by skeletal muscle (Sishi et al. 2011). Given the importance of skeletal muscle function and metabolism in the pathogenesis of obesity, it becomes clear that understanding the capability of (and deficits to) skeletal muscle in those with diet-induced obesity (DIO) to undergo growth and repair is of paramount importance to identify potential avenues to circumvent the loss of skeletal muscle health in the obese condition.

Muscle regeneration following injury is a temporally sensitive and complex series of events with necrosis, phagocytosis, satellite cell (SC) activation/proliferation, de novo myofiber formation, and maturation all overlapping (d’Albis et al. 1988; Hawke et al. 2003; Arnold et al. 2007). A primary contributor to the repair process is the SC population which is activated from its normally quiescent state in response to injury (Anderson 2000). Once activated, the SC begins to proliferate extensively and ultimately undergoes differentiation and fusion to generate new myofibers, or repair existing myofibers (Hawke and Garry 2001). To date, the few studies that have investigated the effect of DIO on muscle repair and SC functionality, though interesting and well executed, have offered conflicting results. These disparities are likely due to variances in diet composition, diet length, and injury type (Hu et al. 2010; Nguyen et al. 2011; Woo et al. 2011). Furthermore, such design differences between and within studies make it difficult to extrapolate findings as detailed analyses of many variables (e.g., derangements to metabolism, insulin sensitivity, and muscle fiber type/composition) that impact the regenerative process in DIO mice remain undefined.

Thus, the purpose of this investigation was to provide a more detailed study of muscle repair and satellite cell functionality in a model system that has been consistently used and thoroughly characterized (Shortreed et al. 2009; Trajcevski et al. 2013; Thomas et al. 2014). We hypothesized that DIO mice subjected to muscle injury would display deficits in the transition from the degenerative to regenerative phase in the muscle repair process, when compared to standard chow-fed mice. Specifically, we hoped to demonstrate a failure for DIO muscle to properly repair itself following injury as a result of alterations to the content and/or function of inflammatory and skeletal muscle cells that participate in muscle regeneration. Furthermore, we wanted to identify whether differences in hepatocyte growth factor (HGF) signaling contributed to any differences in SC function observed between experimental groups.

## Research Methodology

### Animal care

All experimental protocols were approved by the McMaster University Animal Care Committee (AUP #09-08-29) in accordance with the Canadian Council for Animal Care guidelines. Male C57BL/6J mice were obtained from Jackson Laboratories (Bar Harbor, ME). Animals were housed in a temperature- and humidity-controlled facility with a 12/12 h light/dark cycle and had ad libitum access to water and food. At approximately 10 weeks of age, following the diet period of 6 weeks, mice were divided into the following experiments: muscle injury using cardiotoxin (CTX) for analysis of muscle mass, histology and protein expression by immunofluorescence of cross-sections (*N* = 6 per group), single fiber isolation (*N* = 11–27 fibers per CON group, *N* = 18–31 fiber per DIO group), the content of crushed-muscle extract (*N* = 4 animals per group), and proliferation of myofiber-derived myoblasts in culture (*N* = 6–8).

### Diet-induced obesity

Following a 1-week acclimatization period, animals were randomly assigned to either a high-fat diet (TestDiet, cat #58126: energy [kcal/g] from protein [18.3%], fat [60.9%], carbohydrate [20.1%]) or standard mouse chow (LabDiet 5015 Mouse Diet: energy [kcal/g] from protein [20%], fat [25%], carbohydrate [55%]). Mice were maintained on the high fat diet (DIO) or standard chow (CON) for 6 weeks before euthanasia.

### Skeletal muscle injury

Intramuscular injections of CTX (Latoxan, Valence, France; 10 *μ*mol/L) were performed as previously described (Nissar et al. 2012) into the gastrocnemius-plantaris (GP), tibialis anterior (TA), and quadriceps muscles of the left leg of DIO and ND mice. Mice were sacrificed and tissue was collected at 5 or 10 days post-injury.

### Tissue collection

Mice were euthanized by Co_2_ inhalation followed by cervical dislocation. Injured and uninjured TA and GP muscle complexes were excised, weighed, and covered in optimum cutting temperature embedding compound, and frozen in isopentane cooled by liquid nitrogen. Quadriceps muscles from injured and uninjured legs were snap-frozen and stored in −80°C.

### Histochemical and immunofluorescent analyses

Muscles were cut into 8 *μ*m muscle cross-sections and mounted on glass slides to be stained as described below.

#### Picrosirius red for collagen

Muscle sections were immersed in picrosirius red solution (0.1% w/v Direct Red 80 [Sigma 365548] mixed in a saturated aqueous solution of picric acid [Sigma p6744]) for 1 h. Following this, sections were washed with 0.5% glacial acetic acid, dehydrated, cleared, and mounted. This stain enables appropriate quantification of collagen content using fluorescence microscopy, as the collagen retains the fluorescent-red stain, while myofibers appear yellow. The area of red pixels was expressed as percentage of the total injured area per muscle section.

#### Immunofluorescence

Sections were fixed with ice-cold 2% paraformaldehyde for 5 min at 4°C, blocked (PBS with 10% NGS and 1.5% BSA), and incubated with primary antibody overnight at 4°C (rabbit anti-dystrophin, Abcam, 1:200 dilution; rabbit anti-laminin, Abcam, 1:80 dilution; mouse anti-Myosin3, DSHB, used neat; mouse anti-Pax-7, DSHB, neat; mouse anti-MyoD, DAKO, 1:500; chicken anti-laminin, Abcam, 1:250; mouse anti-caveolin 1, Abcam, 1:100; and rat anti-F4/80, AbD Serotec, 1:200). The appropriate Alexa secondary antibody (Invitrogen, Carlsbad, CA) was used for detection of each primary antibody, and nuclei were counterstained using 4,6-diamidino-2-phenylindole (DAPI, 1:10000).

### Image analysis

Images were acquired with a Nikon 90-eclipse microscope (Nikon, Inc., Melville, NY) and analyzed using Nikon Elements software (Nikon, Inc., Melville, NY). Analysis included determination of necrotic regions, Myosin3-positive area, and collagen-positive area in regenerating muscles, and BrdU incorporation on single myofibers. In sections, necrotic fibers were identified by the absence of a distinctive dystrophin ring circling a fiber photographed at 20× magnification. Necrotic fibers also exhibit a disruption and reduction in the expression of laminin surrounding them, as previously reported (Grounds 1998).

### Single myofiber isolation and immunofluorescence

Single myofibers were obtained by collagenase digestion of EDL and peroneus muscles, as previously described (Hawke et al. 2003). Briefly, following collagenase digestion, muscles were triturated with plastic Pasteur pipettes and moved to cell culture dishes with a glass Pasteur pipette. Floating cultures were achieved by coating dishes with 10% normal horse serum prior to addition of plating media (10% normal horse serum, 0.5% chick embryo extract [MP Biomedicals] in low-glucose [1 g/L] Dulbecco’s modified Eagle’s medium [DMEM; Invitrogen]). Single myofibers were incubated in one of three conditions: basal (plating media alone), Recombinant Mouse HGF (R&D, 10 ng/mL), or dimethylsphingosine (DMS, 10 *μ*mol/L) for 45 min, prior to the addition of BrdU to the medium. Satellite cell activation was determined by performing immunoflu-orescence on single myofibers following a 24-h incubation in 5-bromo-2-deoxyuridine (BrdU; Sigma, 10 *μ*mol/L), as previously described (Nissar et al. 2012). The number of BrdU-positive SCs per myofiber were counted on at least 12 myofibers per treatment group as a measure of the functionality of SCs becoming activated by HGF.

### Crushed muscle extract

Crushed muscle extract (CME) was harvested as previously described (Tatsumi et al. 2001) from resting muscle of CON and DIO mice.

### Western blot analysis

About 100 *μ*g of protein derived from crushed muscle extracts and uninjured muscle were run out on a separate acrylamide gel, transferred to PVDF membrane (BioRad, Mississauga, Ontario), blocked with 5% skim milk for 1 h at RT, and then incubated overnight at 4°C with primary Active-HGF antibody (Abcam). A similar western blot protocol was completed for the analysis of c-met (Sigma Aldrich) and myogenin (Novus Biologicals) protein content, using 30 *μ*g of protein from lysates of uninjured and 5 days post-injury muscles, respectively. The appropriate horseradish peroxidase-conjugated secondary antibodies were incubated for 1 h at RT, and the blot was visualized using SuperSignal Chemiluminescent reagent (Thermo Scientific). Images were acquired using a Gel Logic 6000 Pro Imager (Carestream, Rochester, NY) and the area density of each band was analyzed using Adobe Photoshop.

### Primary myoblasts to assay SC proliferative capacity

Primary myoblasts were derived from single myofiber cultures attached to a basement membrane matrix (Matrigel; 1:10 dilution) similar to that previously described (Nissar et al. 2012), with the following exceptions: Following 2 days in plating media, single fibers (1/well in a 24-well plate) were removed and proliferation media (10% FBS, high glucose DMEM) was added to SCs that had migrated off the myofiber. Myoblast number was counted at 0 and 24 h following the addition of proliferation media.

### Statistical analysis

For all experiments, the appropriate *t*-test or two-way ANOVA with Tukey’s post-hoc analysis was performed to analyze differences between DIO and CON groups. Two-way ANOVA was run on data sets with dependent variables measured over time, while one- or two-tailed *t*-tests were carried out on data with only a single comparison. Data are presented as mean ± standard error of the mean with *P* ≤ 0.05 considered significant (denoted by asterisk).

## Results

### Relative muscle mass following injury unaltered with DIO

**Figure 1:**
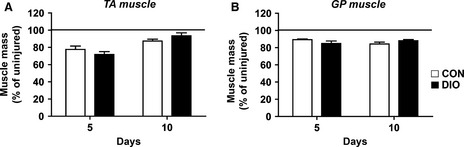
Diet-Induced Obesity does not alter muscle mass 5 and 10 days following CTX injury. Injured muscle mass relative to uninjured contralateral leg 5 or 10 days after CTX injury in the tibialis anterior (TA) muscle and the gastrocnemius/plantaris (GP) muscle complex (*n *=* *6). Significance was determined by two-way ANOVA performed for each muscle type with Bonferroni post-tests, *P *<* *0.05. Control diet (CON, white bars), diet-induced obesity (DIO, black bars).

Absolute muscle mass of the uninjured tibialis anterior (TA) and gastrocnemius/plantaris muscle groups (GP) was previously found to be unaltered after high-fat feeding (Shortreed et al. 2009). Similarly, we report no differences in muscle mass of injured (left) relative to uninjured (right) muscle 5 or 10 days post-injury between diet groups (Fig.1A and B).

### Prolonged necrosis present in muscle following injury with DIO

**Figure 2:**
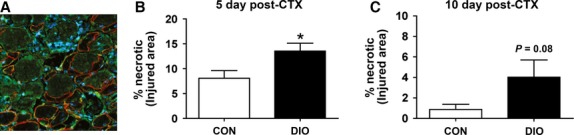
Enhanced necrotic area in CTX-injured skeletal muscle following diet-induced obesity (A) Representative image of CON GP muscle 10 days post CTX stained with laminin (green) and dystrophin (red), which both outline healthy fibers (nuclei stained with DAPI, blue). Arrows indicate necrotic fibers identified by the absence of dystrophin. (B) Percentage area of necrosis in the injured area after 5 days (*n *=* *5). (C) Percentage area of necrosis in the injured area after 10 days (*n *=* *6 CON, 5 DIO). Control diet (CON), diet-induced obesity (DIO). Significance was determined by student’s *t*-test, *P <* *0.05.

In the damaged muscles of DIO mice, the area of necrosis was found to be significantly larger 5 days post-injury compared to that of CON mice, and suggests that the these muscles are unable to appropriately transition from the degeneration to regeneration phase (Fig.2A and B). This greater area of necrosis in muscle from DIO mice remained 10 days post-injury compared to CON (Fig.2C).

### Collagen content, but not macrophage density, is enhanced 5 days post-injury in DIO muscle

**Figure 3:**
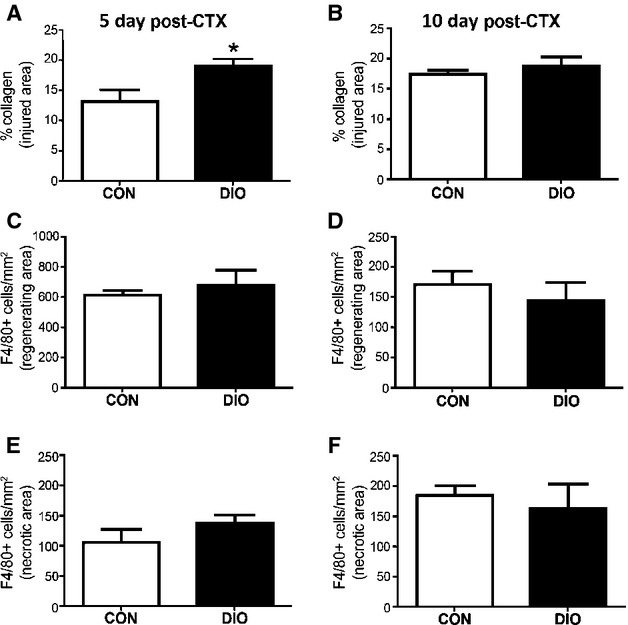
Diet-Induced Obesity increases collagen content during early muscle regeneration. (A and B) Percent collagen content in injured areas at 5 (*n *=* *5 both groups) and 10 days (*n *=* *4 both groups) following injury. (C–F) Macrophage-positive cells (determined by staining for F4/80) in necrotic and regenerating areas at 5 (*n *=* *5 both groups) and 10 days (*n *=* *4 both groups) following injury. Control diet (CON), diet-induced obesity (DIO). Significance was determined by student’s *t*-test, *P <* *0.05.

Consistent with a delay in transitioning to the regenerative phase, injured DIO muscles exhibited a significantly higher collagen content 5 days post-injury (Fig.3A) which was resolved by 10 days post-injury (Fig.3B). Greater collagen content during muscle repair can impair cellular influx (Krause et al. 2013). However, there was no reduction in total (F4/80 positive) macrophage content at 5 or 10 days post-injury with DIO in either the necrotic or regenerating areas (Fig.3C–F).

### Myofiber repair is delayed in muscle with DIO

**Figure 4:**
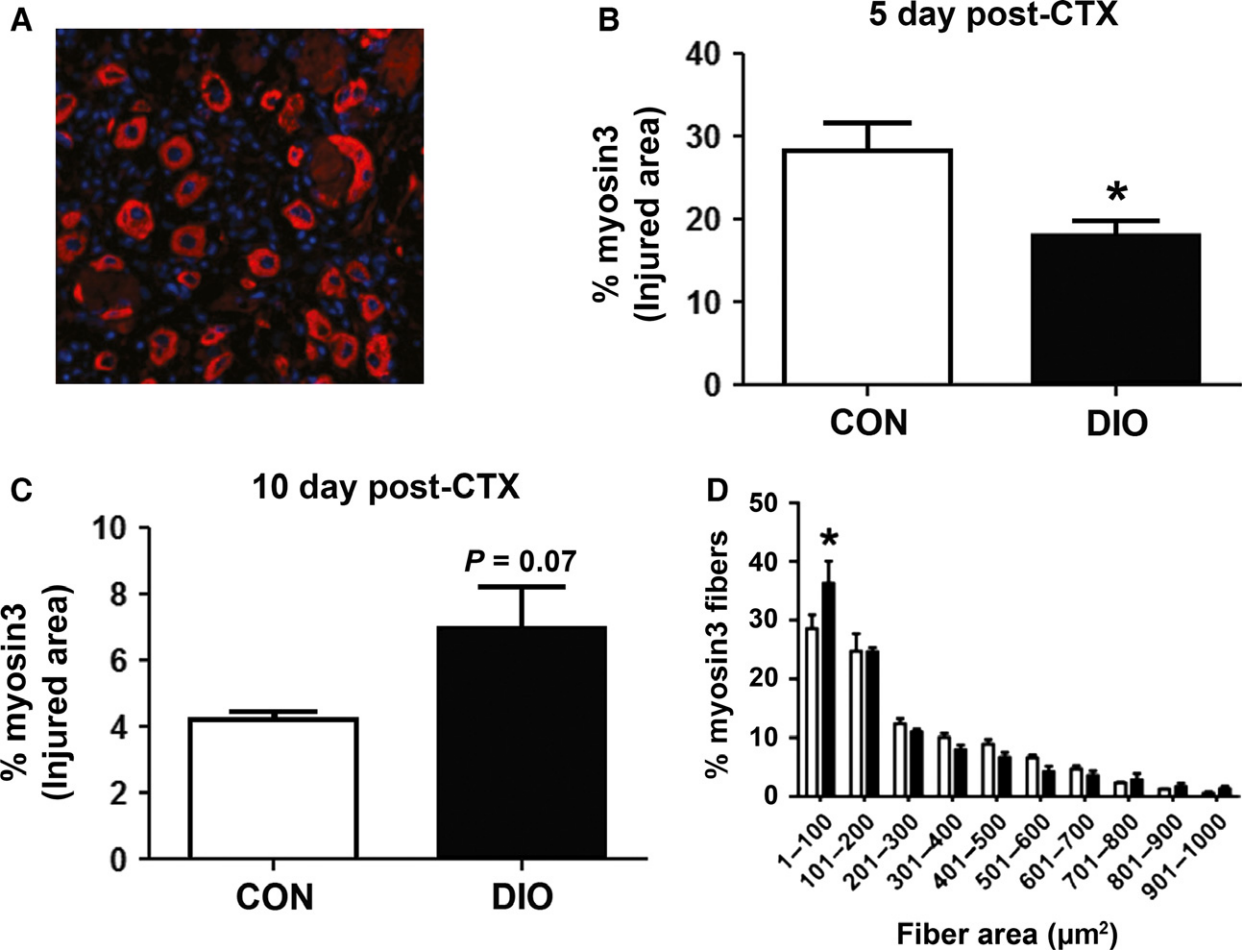
Diet-Induced Obesity delays muscle regeneration in muscle following CTX injury. (A) Representative image of CON GP muscle 5 days after injury stained with Myosin3 (Myh3, red; nuclei stained with DAPI, blue). (B) Percentage area of Myh3 content in the injured area after 5 days (*n *=* *5). (C) Percentage area of Myh3 content in the injured area after 10 days (*n *=* *4). (D) Distribution of Myh3-positive fiber area at 5 days following injury. Control diet (CON), diet-induced obesity (DIO). Significance was determined by student’s *t*-test, *P <* *0.05.

The persistence of necrotic tissue in regenerating DIO muscle indicated a delay in the muscle regenerative process. This led to the quantification of regenerating fiber sizes between diet groups to determine whether alterations in the muscle repair process would be evident. Newly regenerating myofibers express developmental isoforms of myosin heavy chain, including embryonic myosin heavy chain (Myosin3; Fig.4A) prior to expressing more mature forms of myosin, thus rendering Myosin3 an appropriate marker for myofiber regeneration (d’Albis et al. 1988; Krause et al. 2011; Schiaffino et al. 1986). We observed a significantly smaller area of Myosin3 staining in fibers in regenerating DIO muscle at 5 days post-injury compared to CON (Fig.4B). By 10 days post-injury, much of the damaged CON muscle had transitioned to more mature myosin isoforms, while damaged DIO muscle expressed nearly twice as much Myosin3 as the controls (Fig.4C).

To support the conclusion of delayed regeneration, we plotted the cross-sectional areas of Myosin3-positive fibers in CON and DIO muscles at 10 days post-injury. As can be seen in Figure4D, there were significantly smaller Myosin3-positive fibers in DIO muscles compared to CON (Fig.4D), resulting in a leftward shift in newly regenerative fiber size distribution when compared to CON.

### DIO does not alter satellite cell content

**Figure 5:**
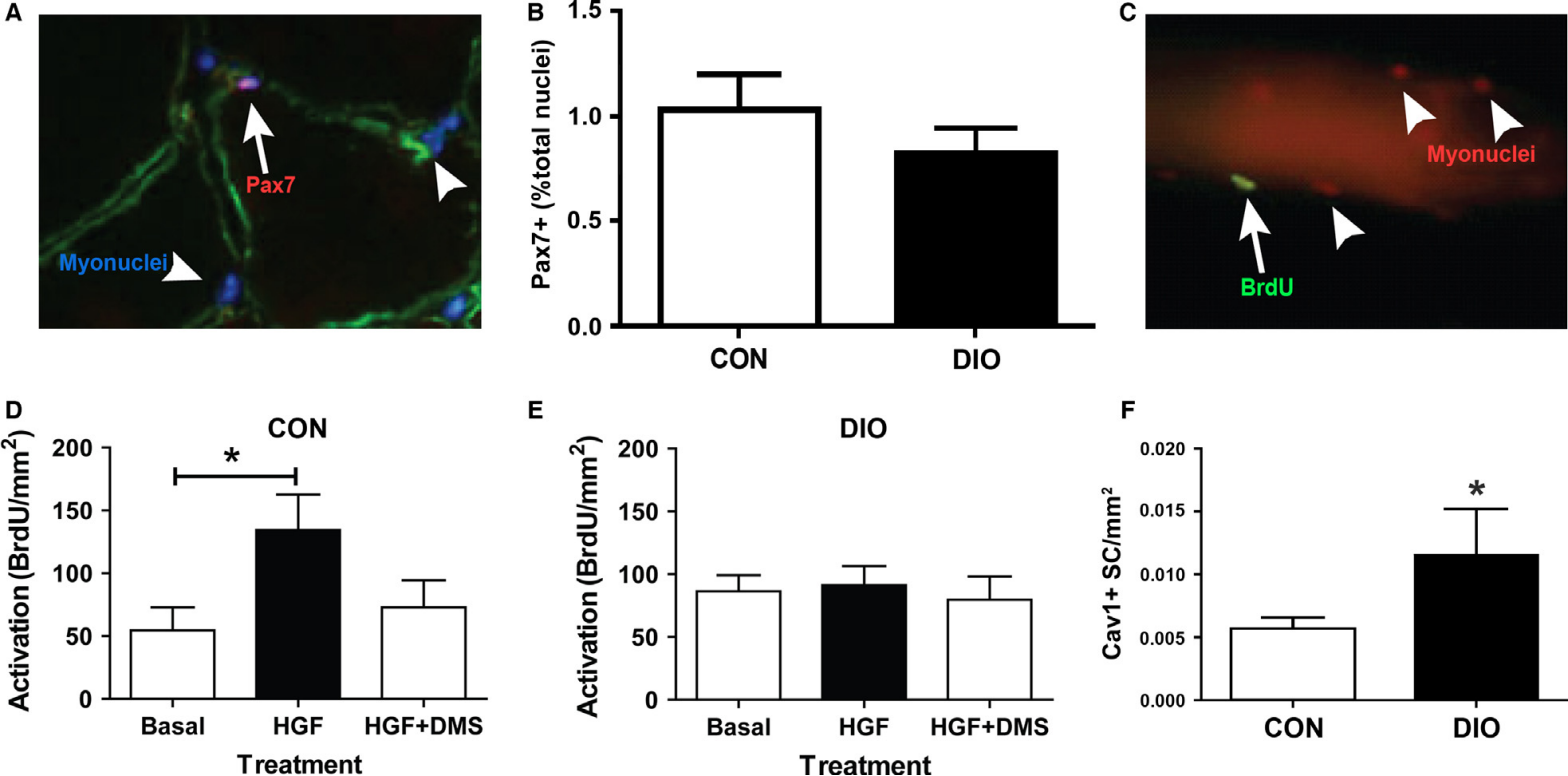
Satellite cell activation is impaired in Diet-Induced Obesity. (A) Representative image of a Pax-7 positive SC (red; nuclei blue) located under the basal lamina (laminin, green). (B) Pax-7 content in uninjured TA muscle (*n *=* *3 CON, 5 DIO). (C) Representative image of a single myofiber with an activated, Bromo-deoxy-uridine (BrdU) positive SC (green; nuclei red). (D and E) Activated SCs (BrdU positive) per fiber relative to fiber area (CON, *n *=* *11–27 fibers/treatment; DIO, *n *=* *18–31 fibers/treatment condition) in response to plating media (Basal), plating media with hepatocyte growth factor (HGF) or plating media with HGF and dimethyl sulphoxide (DMS) in floating cultures of (D) CON and (E) DIO myofibers. Control diet (CON), diet-induced obesity (DIO). (F) Caveolin-1 positive SCs 24 h following CTX injury in GP muscle. (B and F) Significance was determined by student’s *t*-test, *P <* *0.05. (D and E) Significance was determined by a one-way ANOVA with Tukey’s Multiple Comparison post-hoc test, *P <* *0.05.

Considering the importance of satellite cells to regeneration, changes to SC functionality and/or content impact the repair of skeletal muscle (Wang and Rudnicki 2011). There was no difference in SC content of resting muscle between diet groups as determined by evaluating the percentage of Pax7-positive myonuclei (Fig.5A and B). The basal activation state of SCs was investigated by determining the number of MyoD-positive myonuclei in a given area of resting muscle fibers. DIO mice, similar to CON, had very few positive MyoD nuclei (data not shown) demonstrating that there was no increase in basal SC activation with DIO.

### DIO skeletal muscle does not respond to HGF and releases less of it following injury

We next ascertained the ability for SCs from DIO and CON muscles to appropriately respond to external activation stimuli. SC activation has been demonstrated to occur when HGF binds to its receptor, c-Met, on SCs (Anderson and Wozniak 2004). HGF activates sphingosine kinase 1 (SK1), the enzyme that converts sphingomyelin to sphingosine-1-phosphate (S1P), which itself has been shown to initiate proliferation of reserve cells (Duan et al. 2006; Hu et al. 2009). We therefore assessed the ability of HGF to enhance SC activation on freshly isolated myofibers from both CON and DIO mice, as has been previously demonstrated (Wozniak and Anderson 2007; Zhang and Anderson 2014), as well as examined the involvement of S1P in this process. HGF administration (10 ng/mL) successfully activated SCs, promoting their entry into the cell cycle (as demonstrated by BrdU incorporation) on CON muscle fibers (Fig.5C and D) but had no effect on isolated myofibers from DIO muscle (Fig.5E). Co-incubation of myofibers with both HGF and a sphingosine kinase inhibitor (d-erythro-N, N-dimethylsphingosine, DMS; Hu et al. 2009) significantly reduced the number of BrdU-positive SCs on CON myofibers (Fig.5D), but had no effect on the number of BrdU-positive SCs observed on DIO myofibers (Fig.5E). In support of HGF signaling being down-regulated, increased Caveolin-1 protein content, normally down-regulated by HGF following injury (Volonte et al. 2005), was found in DIO compared to CON muscle 24 h post-injury (Fig.5F).

**Figure 6:**
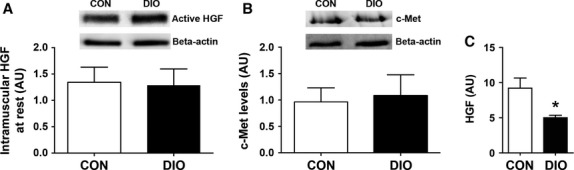
Diet-induced obesity impairs HGF release from injured muscle. (A) Representative blot and graph of intramuscular active HGF in CON and DIO uninjured muscle (*n *=* *4). (B) Representative blot and graph of c-met expression in WT and DIO uninjured muscle (*n *=* *4). (C) Release of active HGF (both *α* and *β* chains) relative to the marker of injury myoglobin in crushed muscle extract (*n *=* *4). Control diet (CON), diet-induced obesity (DIO). Significance was determined by student’s *t*-test, *P <* *0.05.

The increased collagen content evident in regenerating DIO muscle could affect SC activation by impeding the interaction between active HGF and its receptor, c-Met, as has been reported in aged skeletal muscle (Barani et al. 2003; Serrano et al. 2011). No differences in the intramuscular protein content of the active form of HGF or its receptor, c-Met, were observed in uninjured muscle between DIO and CON groups (Fig.6A and B). Furthermore, we determined that there was significantly less HGF released from crushed muscle extracts (CME) from resting DIO mice compared to CON mice (Fig.6C).

### Myoblast proliferative ability is impaired ex vivo and in muscle 5 days after injury with DIO

**Figure 7:**
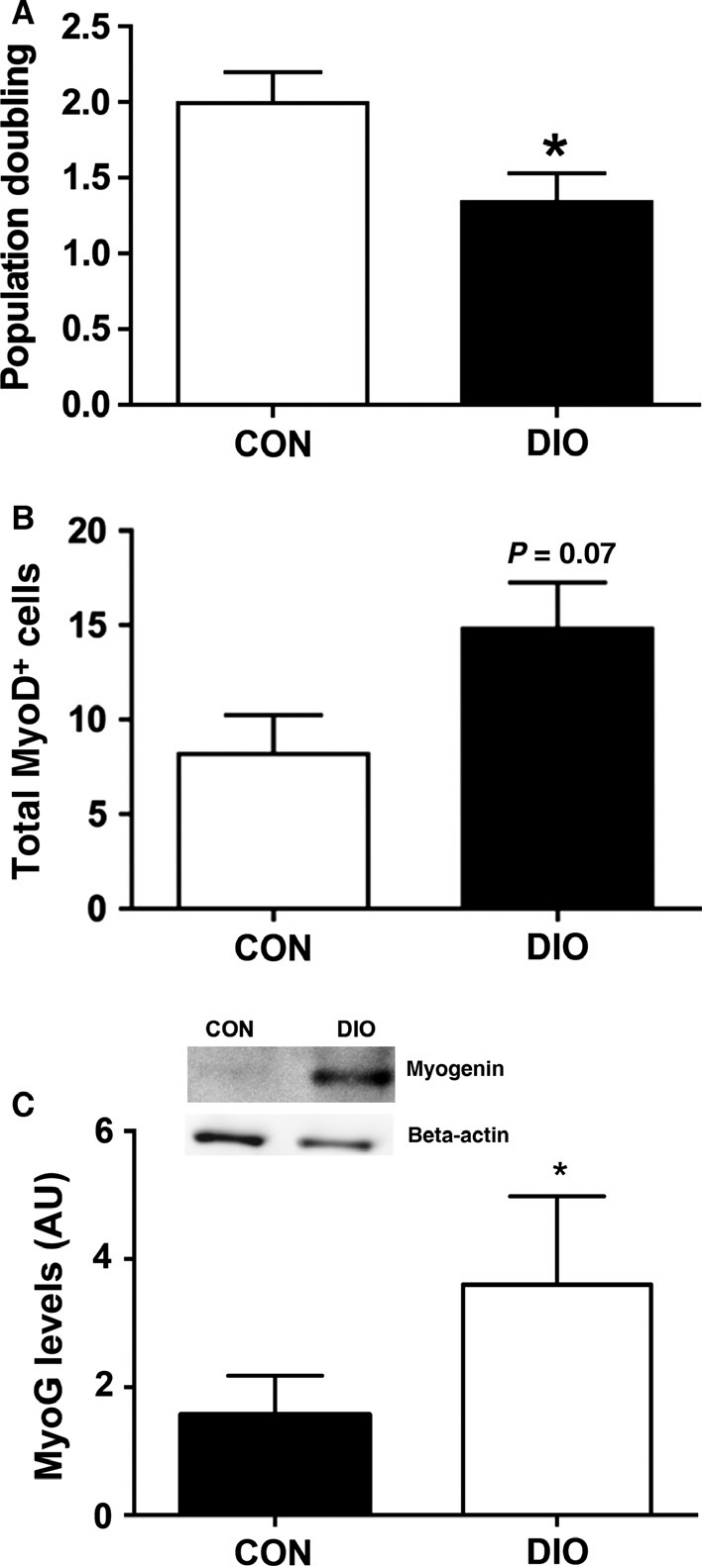
Diet-induced obesity impairs SC proliferation, and attenuates the expression of myogenic markers 5 days following CTX injury. (A) Myoblast proliferation assessed from day 2 to 3 post-harvest in cell culture (*n *=* *6 CON, 8 DIO). (B) MyoD positive cells in TA muscle cross-sections at 5 days post-injury (*n *=* *5). (C) Representative blot and graph of myogenin (MyoG) 5 days following injury (*n *=* *3). Significance was determined by student’s *t*-test, *P <* *0.05.

To assess the impact of lower HGF release from DIO muscles on the proliferative capacity of satellite cells from DIO and CON muscles, primary SC cultures were isolated and permitted to proliferate. Significantly fewer myoblasts were counted in cultures derived from DIO muscles than from cultures of CON muscles (Fig.7A). To support these in vitro results, the number of MyoD-positive cells was quantified in immunofluorescently stained sections from DIO and CON muscles at 5 days post-injury. In healthy control muscles, a peak in myoblast (MyoD-positive) content has been reported at ∼3 days following CTX injury (Yan et al. 2003; Tidball and Villalta 2010) with a reduction in MyoD-positive cells thereafter. We observed a strong trend (*P* = 0.07) toward more MyoD-positive cells in injured DIO muscles compared to CON at 5 days post-injury, supporting the findings of attenuated proliferative capacity in the SC population of DIO muscles (Fig.7B). Western blot analysis of regenerating muscles at 5 days post-injury indicated significantly more Myogenin in DIO muscle compared to regenerating CON muscles (Fig.7C), further corroborating the disruption in SC functionality. Taken together, these results suggest that SCs originating from DIO muscle fail to respond to activating stimuli in a timely fashion, an effect that ultimately results in an attenuated regenerative capacity of the skeletal muscle.

## Discussion

A healthy skeletal muscle mass is integral to the success of efforts to reduce obesity and insulin resistance and, ultimately, in attenuating the progression toward Type 2 Diabetes Mellitus and associated complications. A primary attribute of healthy skeletal muscle is the capacity for efficient maintenance and repair from overuse or injury. In the present study, we observed delayed muscle repair in DIO mice compared to standard-chow controls, as identified by the enhanced presence of necrotic areas and collagen content, as well as a reduction in regenerative fibers in DIO muscle at 5 days post-injury. While a deficit in muscle repair has been noted by some, but not all investigations involving DIO (Hu et al. 2010; Nguyen et al. 2011; Woo et al. 2011), this is the first study to report decrements specifically in muscle SC function and provide evidence for an underlying cause for this impairment. We demonstrate here that DIO induces changes within skeletal muscle that alters HGF signaling, prompting intrinsic impairments in SC activation and proliferation, and a concomitant delay in muscle repair following injury compared to control mice.

Intramuscular injection of cardiotoxin results in significant muscle damage and is an established means to investigate changes to muscle regeneration pathways (Hawke and Garry 2001). Following injury, the muscle will undergo two distinct phases of muscle repair. The degenerative phase comprises removal of damaged cells, accompanied by SC activation and an increase in the collagen matrix. The degenerative phase is followed by the regeneration phase, wherein SCs proliferate and differentiate to repair the damaged muscle (Hawke and Garry 2001; Karalaki et al. 2009). This regenerative phase also involves extensive remodeling of the extracellular matrix (ECM) as new and regenerated myofibers replace the collagen matrix that infiltrated the damaged muscle. Indicators of muscle repair, including morphometric and SC assessments, are often used to detect differences in the repair process (d’Albis et al. 1988; Hirata et al. 2003; Arnold et al. 2007; Sakaguchi et al. 2014). The degenerative phase involves influx of inflammatory cells and necrosis of damaged myofibers which subsides by ∼5 days post-injury. The continued presence of necrotic areas is indicative of a persistent degenerative phase and/or a slowed regenerative process (Serrano et al. 2011). The regenerative phase is characterized by the appearance of numerous small, newly regenerated myofibers (Hawke et al. 2003). As regeneration progresses, the new myofibers increase in size and the expression of myosin heavy chain isoforms transitions from developmental to mature isoforms (Pernitsky et al. 1996; Hawke et al. 2003; Arnold et al. 2007). Previous work reported no difference in muscle fiber area and muscle repair with DIO, as quantified histologically by the number of regenerating fibers and the percent area of regenerating muscle after injury (Nguyen et al. 2011). While this method detected significant reductions in the measured variables for other mouse models of diabetes (ob/ob and db/db) it only detected trends for deficits in mice fed a high-fat diet (60% kcal fat) for 12 weeks. Though their study (Nguyen et al. 2011) may have been underpowered for these specific measurement techniques in the subtler phenotype of the DIO mouse model, it should also be noted that their mice were only 4 weeks old at the time of the high-fat diet commencement. It has previously been reported that young, growing mice fed a high-fat diet do not display many of the impairments exhibited when the diet is initiated in adulthood, likely the result of a much higher metabolic rate in youth (Thomas et al. 2014). Furthermore, quantifying newly regenerating muscle fibers at 5 days post-injury by counting centrally nucleated myofibers in hemotoxylin–eosin-stained sections can be difficult given the very small size of the most recently generated myofibers, especially if there is delayed growth after treatment. In an effort to improve sensitivity to newly regenerating fibers in the present study, we used software-mediated quantification of newly regenerating myofibers through Myosin3 immunofluorescence (Schiaffino et al. 1986). At 5 days post-injury, we observed that DIO muscles had an increased number of very small, newly regenerated myofibers and the relative size of the regenerating area (with respect to the whole area of injury) was smaller than that measured in CON muscles. The presence of persistent, larger necrotic areas within injured DIO muscle was hypothesized to be the result of higher levels of collagen relative to CON muscles, which has been reported to impair macrophage infiltration in T1DM mice (Krause et al. 2013). Our investigations into this hypothesis revealed no difference between diet groups in overall macrophage content within either necrotic or regenerating areas of the damaged muscles. However, while total macrophage content was not different, it is possible that the elevated fibrosis influenced the timely transition of macrophages from a proinflammatory (M1) to an anti-inflammatory (M2) phenotype (Arnold et al. 2007; Brigitte et al. 2010). It is possible that an increased abundance of M1, but not M2, macrophages in DIO muscle may contribute to the lack of HGF production observed in crush-injured DIO muscle, as prior work has identified M2 macrophages as a source of HGF production in injured muscle (Sakaguchi et al. 2014). While we do not directly quantify the two distinct macrophage populations in this study, others have shown that in obese samples, there is an increased presence of inflammatory macrophages (M1) that coincides with an overall increase in total macrophage population (Fink et al. 2014). Furthermore, past work has demonstrated that only 3 weeks of high-fat feeding can promote an excess of M1 macrophages in uninjured muscle (Hong et al. 2009). As such, it seems plausible that a contributing factor to the increased presence of necrosis in DIO muscle is a result of an overall enhanced inflammatory state, thereby attenuating the regenerative process.

Perhaps even more influential to the regenerative process, besides the presence of anti-inflammatory macrophages, is the skeletal muscle satellite cell (SC). Past research has suggested that the cause of impaired regeneration in DIO muscle is primarily associated with reductions in SC content. (Woo et al. 2011). We did not observe a difference in SC content between diet groups in the present study. Rather, we measured significant impairments in SC functionality in DIO muscle compared to control muscle. The attenuation of SC activation in DIO muscles led us to investigate the HGF signaling pathway for its well-characterized role in activating satellite cells from quiescence (Tatsumi et al. 1998). Following injury, HGF is released by injured skeletal muscle and resides in the ECM where it binds to ECM components much like other growth factors (Taipale and Keski-Oja 1997). A significant reduction in HGF release from crushed DIO muscles, compared to CON, was observed in the present study providing a possible mechanism for impaired SC activation. Interestingly, while HGF release from crushed muscle was lower in DIO mice, the total HGF content contained within the muscles did not differ between groups. This would suggest a defective HGF release from sequestration in DIO muscles rather than a reduction in total HGF content. The active form of HGF has been found in skeletal muscle extracellular matrix (Tatsumi and Allen 2004), and while there is no direct evidence relating changes to ECM composition after a period of high-fat feeding with the release of HGF, it is predicted that DIO unfavorably alters ECM composition, deterring the release of HGF from the ECM following muscle injury. Human data examining men subjected to a 10% weight gain induced by overfeeding found evidence for skeletal muscle ECM remodelling, subsequently resulting in skeletal muscle fibrosis (Tam et al. 2014). The results from this human study raise the possibility that the findings of the current study could very well be linked to a failure in the release of HGF from the remodelled ECM in DIO skeletal muscle.

To further elucidate the importance of HGF in DIO SC activation, we examined whether SCs from DIO muscles would respond appropriately to exogenous HGF administration. Exogenous HGF led to a significant increase in CON SCs exiting quiescence and entering the cell cycle (BrdU-positive) as expected. However, this same protocol was completely ineffective at activating SCs on myofibers isolated from DIO muscles. Components of the ECM remain intact following single fiber isolation (Bischoff 1986; Meyer and Lieber 2011), and thus the lack of response observed in DIO single fibers treated with exogenous HGF might be attributed to the inability of HGF to properly communicate with SCs due to an altered/thickened ECM. The absence of HGF-mediated in vitro activation of SCs on single DIO muscle fibers prompted further investigation of the HGF/c-met signaling pathway. Caveolin-1 (Cav1) is a member of the caveolin family of scaffolding proteins that play a role in the formation of caveolae in the plasma membrane (Okamoto et al. 1998; Parton and Simons 2007). Cav1 is expressed on SCs and aids in maintaining the quiescent state (Volonte et al. 2005). Following muscle injury, Cav1 is down-regulated by HGF, thus stimulating the progression of SCs through myogenesis (Volonte et al. 2005). While down-regulation of Cav1 is evident in CON muscle at 24-h post-injury, this same response is not evident in injured DIO muscle, and supports the impairment of HGF-mediated SC activation and proliferation. These results coincide with the delay in Myosin3 expression, a marker of differentiation, in DIO muscle at 5 days post-injury compared to controls. Overall, the results of the present study indicate that the HGF pathway is not being activated appropriately, and is predicted to be a result of aberrant ECM remodelling in DIO muscle.

The present study demonstrates a novel mechanism for decrements in the repair of DIO skeletal muscle. It should also be noted that HGF administered to myotubes in culture enhanced glucose uptake and metabolism (Perdomo et al. 2008). Moreover, glucose uptake and metabolism are impaired in obese, insulin-resistant states despite concomitant elevations in adipose and serum HGF levels (Rehman et al. 2003). Taken together with our present findings, we hypothesize that a common mechanism, defective HGF signaling, may be contributing to the impairments in SC functionality, muscle repair and skeletal muscle metabolism. Such findings are integral to the study of skeletal muscle health in DIO as it highlights the significance of the SC to skeletal muscle growth and repair in DIO. By hindering SC function, as identified through increases in fibrosis and impairments in activation signaling, SCs are unable to function appropriately, and over time, could contribute to the decline in skeletal muscle health of obese individuals. This deficit in skeletal muscle health and function would negatively impact physical capacities, further perpetuating the obese condition and accelerating the onset of additional co-morbidities due to the pivotal role of skeletal muscle in overall health. This condition may worsen with increasing age as other factors also impair satellite cell function, ultimately leading to the state of sarcopenic obesity (Stenholm et al. 2008).

## Conflict of Interest

None declared.

## References

[b1] Akhmedov D, Berdeaux R (2013). The effects of obesity on skeletal muscle regeneration. Front. Physiol.

[b2] d’Albis A, Couteaux R, Janmot C, Roulet A, Mira JC (1988). Regeneration after cardiotoxin injury of innervated and denervated slow and fast muscles of mammals. Myosin isoform analysis. Eur. J. Biochem.

[b3] Anderson JE (2000). A role for nitric oxide in muscle repair: nitric oxide-mediated activation of muscle satellite cells. Mol. Biol. Cell.

[b4] Anderson JE, Wozniak AC (2004). Satellite cell activation on fibers: modeling events in vivo–an invited review. Can. J. Physiol. Pharmacol.

[b5] Arnold L, Henry A, Poron F, Baba-Amer Y, van Rooijen N, Plonquet A (2007). Inflammatory monocytes recruited after skeletal muscle injury switch into antiinflammatory macrophages to support myogenesis. J. Exp. Med.

[b6] Barani AE, Durieux AC, Sabido O, Freyssenet D (2003). Age-related changes in the mitotic and metabolic characteristics of muscle-derived cells. J. Appl. Physiol.

[b7] Bischoff R (1986). Proliferation of muscle satellite cells on intact myofibers in culture. Dev. Biol.

[b8] Brigitte M, Schilte C, Plonquet A, Baba-Amer Y, Henri A, Charlier C (2010). Muscle resident macrophages control the immune cell reaction in a mouse model of notexin-induced myoinjury. Arthritis Rheum.

[b9] Duan HF, Qu CK, Zhang QW, Yu WM, Wang H, Wu CT (2006). Shp-2 tyrosine phosphatase is required for hepatocyte growth factor-induced activation of sphingosine kinase and migration in embryonic fibroblasts. Cell. Signal.

[b10] Fink LN, Costford SR, Lee YS, Jensen TE, Bilan PJ, Oberbach A (2014). Pro-inflammatory macrophages increase in skeletal muscle of high fat-fed mice and correlate with metabolic risk markers in humans. Obesity.

[b11] Grounds MD (1998). Age-associated changes in the response of skeletal muscle cells to exercise and regeneration. Ann. N. Y. Acad. Sci.

[b12] Hawke TJ, Garry DJ (2001). Myogenic satellite cells: physiology to molecular biology. J. Appl. Physiol.

[b13] Hawke TJ, Meeson AP, Jiang N, Graham S, Hutcheson K, DiMaio JM (2003). p21 is essential for normal myogenic progenitor cell function in regenerating skeletal muscle. Am. J. Physiol. Cell Physiol.

[b14] Hirata A, Masuda S, Tamura T, Kai K, Ojima K, Fukase A (2003). Expression profiling of cytokines and related genes in regenerating skeletal muscle after cardiotoxin injection: a role for osteopontin. Am. J. Pathol.

[b15] Hong EG, Hwi JK, Cho YR, Kim HJ, Ma Z, Yu TY (2009). Interleukin-10 prevents diet-induced insulin resistance by attenuating macrophage and cytokine response in skeletal muscle. Diabetes.

[b16] Hu W, Bielawski J, Samad F, Cowart AH, Merrill LA (2009). Palmitate increases sphingosine-1-phosphate in C2C12 myotubes via upregulation of sphingosine kinase message and activity. J. Lipid Res.

[b17] Hu Z, Wang H, Lee IH, Modi S, Wang X, Du J (2010). PTEN inhibition improves muscle regeneration in mice fed a high-fat diet. Diabetes.

[b18] Karalaki M, Fili S, Philippou A, Koutsilieris M (2009). Muscle regeneration: cellular and molecular events. In Vivo.

[b19] Knoblauch MA, O’Connor DP, Clarke MS (2013). Obese mice incur greater myofiber membrane disruption in repsonse to mechanical load compared with lean mice. Obesity.

[b2000] Krause MP, Moradi J, Nissar AA, Riddell MC, Hawke TJ (2011). Inhibition of plasminogen activator inhibitor-1 restores skeletal muscle regeneration in untreated type 1 diabetic mice. Diabetes.

[b20] Krause MP, Al-Sajee D, D’Souza DM, Rebalka IA, Moradi J, Riddell MC (2013). Impaired macrophage and satellite cell infiltration occurs in a muscle-specific fashion following injury in diabetic skeletal muscle. PLoS ONE.

[b21] Meyer GA, Lieber RL (2011). Elucidation of extraceullar matrix mechanics from muscle fibers and fiber bundles. J. Biomech.

[b22] Nguyen M-H, Cheng M, Koh TJ (2011). Impaired muscle regeneration in ob/ob and db/db mice. Sci. World J.

[b23] Nissar AA, Zemanek B, Labatia R, Atkinson DJ, van der Ven PFM, Furst DO (2012). Skeletal muscle regeneration is delayed by reduction in Xin expression: consequence of impaired satellite cell activation?. Am. J. Physiol.

[b24] Okamoto T, Schlegel A, Scherer PE, Lisanti MP (1998). Caveolins, a family of scaffolding proteins for organizing “preassembled signaling complexes” at the plasma membrane. J. Biol. Chem.

[b25] Parton RG, Simons K (2007). The multiple faces of caveolae. Nat. Rev. Mol. Cell Biol.

[b26] Perdomo G, Martinez-Brocca MA, Bhatt BA, Brown NF, O’Doherty RM, Garcia-Ocaña A (2008). Hepatocyte growth factor is a novel stimulator of glucose uptake and metabolism in skeletal muscle cells. J. Biol. Chem.

[b27] Pernitsky AN, McIntosh LM, Anderson JE (1996). Hyperthyroidism impairs early repair in normal but not dystrophic mdx mouse tibialis anterior muscle. An in vivo study. Biochem. Cell Biol.

[b28] Rehman J, Considine RV, Bovenkerk JE, Li J, Slavens CA, Jones RM (2003). Obesity is associated with increased levels of circulating hepatocyte growth factor. J. Am. Coll. Cardiol.

[b29] Sakaguchi S, Shono J, Suzuki T, Sawano S, Anderson JE, Do MK (2014). Implication of anti-inflammatory macrophages in regenerative moto-neuritogenesis: promotion of myoblast migration and neural chemorepellent semaphorin 3A expression in injured muscle. Int. J. Biochem. Cell Biol.

[b30] Salvadori A, Fanari S, Ruga S, Brunani A, Longhini E (1992). Creatine kinase and creatine kinase-MB isoenzyme during and after exercise testing in normal and obese young people. Chest.

[b31] Schiaffino S, Gorza L, Sartore S, Saggin L, Carli M (1986). Embryonic myosin heavy chain as a differentiation marker of developing human skeletal muscle and rhabdomyosarcoma. A monoclonal antibody study. Exp. Cell Res.

[b32] Serrano AL, Mann CJ, Vidal B, Ardite E, Perdiguero E, Munoz-Canoves P (2011). Cellular and molecular mechanisms regulating fibrosis in skeletal muscle repair and disease. Curr. Top. Dev. Biol.

[b33] Shortreed KE, Krause MP, Huang JH, Dhanani D, Moradi J, Ceddia RB (2009). Muscle-specific adaptations, impaired oxidative capacity and maintenance of contractile function characterize diet-induced obese mouse skeletal muscle. PLoS ONE.

[b34] Sishi B, Loos B, Ellis B, Smith W, du Toit EF, Engelbrecht AM (2011). Diet-induced obesity alters signalling pathways and induces atrophy and apoptosis in skeletal muscle in a prediabetic rat model. Exp. Physiol.

[b35] Stenholm S, Harris TB, Rantanen T, Visser M, Kritchevsky SB, Ferrucci L (2008). Sarcopenic obesity: definition, cause and consequences. Curr. Opin. Clin. Nutr. Metab. Care.

[b36] Taipale J, Keski-Oja J (1997). Growth factors in the extracellular matrix. FASEB J.

[b37] Tam CS, Covington JD, Bajpeyi S, Tchoukalova Y, Burk D, Johannsen DL (2014). Weight gain reveals dramatic increases in skeletal muscle extracellular matrix remodelling. J. Clin. Endocrinol. Metab.

[b38] Tatsumi R, Allen RE (2004). Active hepatocyte growth factor is present in skeletal muscle extracellular matrix. Muscle Nerve.

[b39] Tatsumi R, Anderson JE, Nevoret CJ, Halevy O, Allen RE (1998). HGF/SF is present in normal adult skeletal muscle and is capable of activating satellite cells. Dev. Biol.

[b40] Tatsumi R, Sheehan SM, Iwasaki H, Hattori A, Allen RE (2001). Mechanical stretch induces activation of skeletal muscle satellite cells in vitro. Exp. Cell Res.

[b41] Thomas MM, Trajcevski KE, Coleman SK, Jiang M, Di Michele J, O’Neill HM (2014). Early oxidative shifts in mouse skeletal muscle morphology with high-fat diet consumption do not lead to functional improvements. Physiol Rep.

[b42] Tidball JG, Villalta SA (2010). Regulatory interactions between muscle and the immune system during muscle regeneration. Am. J. Physiol. Regul. Integr. Comp. Physiol.

[b43] Trajcevski KE, O’Neill HM, Wang DC, Thomas MM, Al-Sajee D, Steinberg GR (2013). Enhanced lipid oxidation and maintenance of muscle insulin sensitivity despite glucose intolerance in a diet-induced obesity mouse model. PLoS ONE.

[b44] Volonte D, Liu Y, Galbiati F (2005). The modulation of caveolin-1 expression controls satellite cell activation during muscle repair. FASEB J.

[b45] Wang YX, Rudnicki MA (2011). Satellite cells, the engines of muscle repair. Nat. Rev. Mol. Cell Biol.

[b46] Wolfe RR (2006). The underappreciated role of muscle in health and disease. Am. J. Clin. Nutr.

[b47] Woo M, Isganaitis E, Cerletti M, Fitzpatrick C, Wagers AJ, Jimenez-Chillaron J (2011). Early life nutrition modulates muscle stem cell number: implications for muscle mass and repair. Stem Cells Dev.

[b48] Wozniak AC, Anderson JE (2007). Nitric oxide-dependence of satellite stem cell activation and quiescence on normal skeletal muscle fibers. Dev. Dyn.

[b49] Yan Z, Choi S, Liu X, Zhang M, Schageman JJ, Lee SY (2003). Highly coordinated gene regulation in mouse skeletal muscle regeneration. J. Biol. Chem.

[b50] Zhang H, Anderson JE (2014). Satellite cell activation and populations on single muscle-fiber cultures from adult zebrafish (Danio rerio). J. Exp. Biol.

[b51] Zurlo F, Larson K, Bogardus C, Ravussin E (1990). Skeletal muscle metabolism is a major determinant of resting energy expenditure. J. Clin. Invest.

